# Exploring determinants of sex and family history-based disparity in type 2 diabetes mellitus prevalence among clinical patients

**DOI:** 10.1186/s12889-024-18170-0

**Published:** 2024-03-04

**Authors:** Fahad Abdulaziz Alrashed, Muhammad Iqbal, Abdulrahman M Alsubiheen, Tauseef Ahmad

**Affiliations:** 1 Department of Cardiac Sciences, College of Medicine, King Saud University, 11472 Riyadh, P.O. Box 7805, Saudi Arabia; 2 Department of Physiology, College of Medicine, King Saud University, 11472 Riyadh, Saudi Arabia; 3 Rehabilitation Sciences Department, College of Applied Medical Sciences, King Saud University (KSU), 11433 Riyadh, P.O. Box 10219, Saudi Arabia; 4 Department of Medical education, College of Medicine, King Saud University, 11472 Riyadh, P.O. Box 7805, Saudi Arabia

**Keywords:** Type 2 diabetes Mellitus, Non-diabetic, Family history of diabetes, Diabetes comorbidity, Prevalence of DM, Saudi Arabia

## Abstract

**Background:**

Type 2 diabetes mellitus represents a multifaceted disorder characterized by intricate pathophysiological mechanisms, encompassing diminished insulin secretion, augmented hepatic glucose production, and heightened insulin resistance. This study aims to assess the sex (Male and Female only) and family history-based differences in the prevalence of T2DM and explore the determinants contributing to this disparity among clinical patients.

**Subjects and methods:**

The study encompassed a diverse pool of clinical patients, encompassing both individuals with diabetes and those without the condition, who had previously sought medical attention for clinical checkups at healthcare centers. The collected data included essential parameters such as blood pressure, weight, height, smoking habits, educational background, and physical activity levels. To ensure methodological rigor and data accuracy, blood pressure measurements adhered to the stringent guidelines set forth by the World Health Organization.

**Results:**

Participants of the present study reported diabetes, among which notable findings emerged regarding health indicators. It was observed that the prevalence of high blood pressure, obesity, and high blood cholesterol exhibited a statistically significant increase among the female participants, underscoring the sex-based disparities in these health parameters. The male population aged 60 or older, the presence of a family history of DM accentuated this risk, resulting in a striking 3.1 times higher prevalence compared to females, who exhibited a 2.4 times higher risk (OR = 2.4, *p* = 0.0008). This intriguing relationship between diabetes and cholesterol levels was not limited to sex. Both male (OR = 2.47) and female (OR = 2.1) diabetes patients displayed highly significant associations with cholesterol levels. The risk of T2DM was significantly associated with triglycerides in both sexes (1.58 times higher in males, and 1.71 times higher in females).

**Conclusions:**

The significance of hypertension as a comorbidity in T2DM, highlighting sex-specific associations and the potential impact of a family history of diabetes on blood pressure. Our findings emphasize the importance of considering lipid profiles, obesity, and their sex-specific associations when assessing and managing diabetes risk. Comprehensive diabetes care should include strategies for lipid control, weight management, and cardiovascular risk reduction, tailored to the individual’s sex and specific risk profile.

**Supplementary Information:**

The online version contains supplementary material available at 10.1186/s12889-024-18170-0.

## Introduction

Type 2 diabetes mellitus (T2DM) represents a multifaceted disorder characterized by intricate pathophysiological mechanisms, encompassing diminished insulin secretion, augmented hepatic glucose production, and heightened insulin resistance [1–3]. T2DM stands as the predominant form of diabetes, accounting for approximately 90 to 95% of all documented diabetes cases [4]. A study report, the global population of individuals aged 18 years and older affected by T2DM reached a staggering 422 million, translating to a prevalence of approximately 8.5%. Notably, the highest prevalence of T2DM is concentrated in middle and low-income countries, where the incidence continues to surge relentlessly [5, 6]. A study publish in Iran, a developing nation, the prevalence of diabetes among individuals aged 40 and above exceeds a noteworthy 24%, underscoring the substantial burden of T2DM within this demographic [7, 8]. These statistics underscore the urgency of addressing T2DM as a burgeoning global health concern with profound implications for both developed and developing regions. The absence of a definitive treatment for T2DM coupled with its potentially fatal consequences has positioned it as one of the most formidable diseases in the medical landscape. T2DM not only stands as a pivotal risk factor for cardiovascular diseases but also serves as the predominant catalyst for the development of debilitating microvascular complications. The risk of cardiovascular disease, stroke, and peripheral vascular disease is substantially increased in people with T2DM [9, 10]. T2DM and Cardiovascular diseases (CVD) were linked by complex mechanisms that include insulin resistance, inflammation, dyslipidemia, and endothelial dysfunction. These factors collectively contribute to accelerated atherosclerosis and an elevated risk of adverse cardiovascular events [11]. Renal complications, particularly diabetic nephropathy, were common in individuals with T2DM. Diabetic nephropathy is a leading cause of end-stage renal disease (ESRD), necessitating dialysis or renal transplantation [12]. These complications encompass severe outcomes such as limb amputation, vision impairment leading to blindness, and the insidious progression towards chronic renal failure, all of which can profoundly diminish the overall quality of life for affected individuals [1].

According to International Diabetes Federation (IDF) region-wise data, diabetes prevalence ranged from 8 to 22% among adults aged 20–79 years in Gulf countries. Saudi Arabia had the highest number of diabetes-related deaths, while Kuwait had the highest prevalence (22%) [13]. In recent decades, Saudi Arabia has witnessed an alarming increase in the number of people with diabetes due to rapid epidemiological changes, urbanization, unhealthy diets, and reduced physical activity [14]. Diabetes also has a significant economic impact. According to estimates, Saudi Arabia spent 17 billion Riyals on diabetes-related treatment in 2014, and people with diabetes were ten times more likely to have public medical healthcare expenditures than those without diabetes [15, 16].

It is imperative to recognize that these intricate and pervasive complications exert a substantial economic burden on both healthcare systems and society at large [17, 18]. This economic strain arises from the extensive resources required for the management and treatment of T2DM and its associated complications. Therefore, understanding and addressing the multifaceted challenges posed by T2DM is not only a medical imperative but also an economic necessity for healthcare systems and society as a whole. It represents a global health challenge, and its prevalence has been steadily rising, notably in Saudi Arabia. The impact of T2DM is multifaceted, and recent research has begun to explore how sex and family history may influence not only the prevalence of T2DM but also its comorbidity patterns and associated determinants among clinical patients in Saudi Arabia [19–21].

Saudi Arabia has witnessed significant socioeconomic development and urbanization in recent decades, with concomitant changes in lifestyle, including dietary habits, sedentary behaviors, and stress levels. These shifts have contributed to an elevated risk of developing T2DM among the Saudi population [1, 3, 22]. Furthermore, family history of T2DM and genetic predisposition were acknowledged risk factors that underscore the importance of understanding the influence of family history on the prevalence and comorbidity of T2DM [21, 23].

It is imperative to investigate sex-based variations in T2DM prevalence. Family history of T2DM can provide valuable insights into the genetic and environmental determinants of the disease, potentially elucidating its comorbidity with other health conditions.

To address these pressing questions, this study seeks to assess sex and family history-based differences in the prevalence of T2DM and explore how these factors may contribute to the comorbidity patterns of the disease among clinical patients in Saudi Arabia. This study aims to assess the sex and family history-based differences in the prevalence of T2DM and explore the determinants contributing to this disparity among clinical patients in Saudi Arabia.

## Subjects and methods

### Study design and setting

The research encompassed a diverse pool of clinical patients, encompassing both individuals with diabetes and those without the condition, who had previously sought medical attention for clinical checkups at healthcare centers. The study was conducted over a span of seven months, ranging from January 2023 to July 2023, and data were meticulously collected from three primary healthcare centers in the Riyadh, Saudi Arabia. The study population was meticulously defined as individuals aged 18 years or older, ensuring a focused examination of T2DM and its determinants. Pregnant women, individuals with communication limitations, and those grappling with mental health issues were thoughtfully excluded from the study, thus ensuring the reliability and validity of the findings. We used random digit table in our study. The table contains the medical records of patients with diabetes from several hospitals. Once the table generated the random numbers, members of our research team cross-referenced them with the list of potential participants. Participants were selected for the sample if their identification number matched the random number. Members of our team continued the process until the desired sample size was achieved. Participation in the study was entirely voluntary, safeguarding the ethical principles of informed consent and autonomy. Prior to engaging in the study, participants willingly provided their informed consent by signing a consent form. To maintain the highest standards of data collection and reliability, all study participants underwent individual interviews conducted by experienced members of the research team. The investigator or a qualified designee meticulously assessed each patient’s eligibility, reviewing both inclusion and exclusion criteria. This was done to ensure their suitability for participation. Patients were identified as having the target disease based on documented diagnoses. The study inclusion criteria included patients who were required to have received a prior T2DM diagnosis from a physician before enrolling. Participants needed to be eighteen years old at their enrollment visit. Patients must have visited the study site at least once between January 2, 2023, and July 30, 2023. Additionally, individuals were eligible if they had a clinical record at the healthcare center. Exclusion criteria for the study included: individuals with gestational diabetes mellitus. Patients with other forms of secondary diabetes were also not eligible. Those unable to provide informed consent or effectively communicate, including mental illness patients, were excluded from participation.

### Data collection

To obtain approval from the IRB, a questionnaire developed from an exhaustive literature review was submitted to the Ethics Committee for approval. This version contained twenty items, which were thoroughly discussed among a panel of three family medicine consulting team members. After two meetings, thirteen items were agreed upon for this study. A team of highly trained researchers conducted comprehensive health assessments directly at the research site, meticulously adhering to a standardized data collection protocol. These assessments encompassed not only questionnaire-based interviews but also a range of vital physical measurements. During the patients’ clinic visit, our team members asked patients about their smoking habits, education, and physical activity levels without using any instruments. Additionally, pertinent medical records from the hospital archives included participants’ ages and their total cholesterol and triglyceride levels. To further evaluate participants’ health status, individual Body Mass Index (BMI) values were calculated by dividing each participant’s weight in kilograms by the square of their height in meters. To ensure methodological rigor and data accuracy, blood pressure measurements adhered to the stringent guidelines set forth by the World Health Organization/International Society of Hypertension. Blood pressure was measured three times on the right arm, utilizing standardized mercury sphygmomanometers, thereby adhering to recognized standards of measurement accuracy. It is worth noting that the researchers who conducted these assessments were final-year medical students enrolled in MBBS programs, having received uniform and comprehensive training to guarantee consistency and reliability in data collection procedures. In cases where missing details or errors were identified, additional interviews or examinations were conducted to rectify and enhance the dataset’s integrity. Furthermore, meticulous attention was given to standardizing all measurement instruments, ensuring that the data collected would meet the highest standards of accuracy and reliability in line with the study’s scientific objectives and research questions.

### Instrument and data setting

An Arabic and English bilingual questionnaire was developed following a comprehensive review of previous studies and guidelines. The initial section of data collection focused on gathering crucial sociodemographic information from all participants. This comprehensive questionnaire covered an array of sociodemographic variables, including age, sex, ethnicity, marital status, educational attainment, occupation, family medical history, smoking habits, and any prior hypertension history. These data points served as essential foundational insights into participants’ backgrounds and risk factors. The second segment of the data collection process delved into detailed anthropometric measurements. These measurements encompassed vital health indicators such as weight, height, and BMI, providing valuable insights into participants’ overall health status. Additionally, the comprehensive assessment extended to cardiovascular risk factors, including fasting blood sugar levels, blood lipid profiles (cholesterol and triglyceride levels), and systolic and diastolic blood pressure (BP) measurements. Creatinine levels, a significant marker of kidney function, were also included in this section to provide a more comprehensive overview of the participants’ health status.

### Statistical analysis

We employ Microsoft Excel as our data input and analysis tool for comprehensive data management. For robust statistical analysis, we utilized SPSS version 24.0 by IBM, headquartered in Armonk, NY, USA. In addition to calculating prevalence, we computed 95% confidence intervals to enhance our estimation precision. To elucidate the associations between specific outcomes and the variables under consideration, we harnessed Pearson’s chi-square test and odds ratios (OR) to quantify risk factors and awareness associations. Throughout this study, we maintained a rigorous significance level of *p* < 0.05 to ensure our findings.

## Results

The study encompassed a dataset comprising 598 participants, each included in our analysis. In Table 1, we meticulously detail the sociodemographic attributes of this diverse population, along with a comprehensive examination of the prevalence of T2DM. Within this cohort of 598 patients, a sex distribution was observed, with 317 (53.0%) representing male patients and 281 (47.0%) representing female patients. Notably, among the male participants, 23% were identified as individuals with diabetes. A slightly higher proportion of 31.3% exhibited signs of diabetes among the female participants. The study revealed that the highest prevalence of Type 2 Diabetes Mellitus (T2DM) occurred among patients aged 60 years and above, with a substantial prevalence rate of 44.3% in this age group. Notably, T2DM prevalence was markedly higher among those who were illiterate or had completed only primary education (*p* = 0.07). In the current study an estimated 88.1% of the surveyed population lived in urban areas. Interestingly, despite the urban majority, the prevalence of T2DM was noticeably elevated among the rural demographic, standing at 36.6%, compared to 25.6% among their urban counterparts. It’s worth noting that while this difference in prevalence rates was notable, our statistical analysis did not identify it as statistically significant (*P* = 0.41). Within our study cohort, 15.2% of participants were smokers. Moreover, 62.2% reported a diabetes family history. Remarkably, a statistically significant correlation (*p* = 0.004) emerged between individuals with a family history of diabetes and the prevalence of diabetes, with 28.8% of these participants being affected. Furthermore, our analysis revealed that 18.9% of the participants engaged in regular physical activity. Interestingly, a majority of these physically active individuals did not have diabetes; however, 33.6% of those who maintained an active lifestyle also exhibited signs of diabetes. We meticulously monitored the blood pressure of 32.8% of the study participants and assessed cholesterol levels in 35.3% of the cohort. Among the participants in the current study, only 15.2% reported that they smoked. Additionally, we identified that a relatively small subset, comprising only 6.2% of participants (37 individuals), met the criteria for obesity. Remarkably, within this group, 17% were diagnosed with T2DM (Table 1).

**Table 1:** Prevalence of type 2 diabetes socio-demographic characteristics of the respondents

Items	Categories	n(%)	T2DM		*p*-value
			Yes - n(%)	No- n(%)	
Gender	Male	317(53.0)	73(23.0)	244(77.0)	0.03
	Female	281(47.0)	88(31.3)	193(68.7)	
Age	18–39	238(39.8)	51(21.4)	187(78.6)	0.17
	40–49	162(27.1)	37(22.8)	125(77.2)	
	50–59	137(22.9)	46(33.6)	91(66.4)	
	60 or older	61(10.2)	27(44.3)	34(55.7)	
Education Level	illiterate	18(3.0)	9(50.0)	9(50.0)	0.07
	Primary schooling	67(11.2)	23(34.3)	44(65.7)	
	Secondary Schooling	197(32.9)	61(31.0))	136(69.0)	
	Graduate	208(39.8)	48(23.1)	160(76.9)	
	Post-graduate	78(13.0)	20(25.6)	58(74.4)	
Residency	Urban	527(88.1)	135(25.6)	392(74.4)	0.41
	Rural	71(11.9)	26(36.6)	45(63.4)	
Family history of DM	Yes	372(62.2)	107(28.8)	265(71.2)	0.004
Physically active	Yes	113(18.9)	38(33.6)	75(66.4)	0.03
High blood pressure	Yes	196(32.8)	87(44.4)	109(55.6)	0.07
Cholesterol	Yes	211(35.3)	73(34.6)	138(65.4)	0.001
smoking	Yes	91(15.2)	38(41.8)	53(58.2)	0.09
BMI	underweight	71(11.9)	11(15.5)	60(84.5)	0.28
	Normal	273(45.7)	64(23.4)	209(76.6)	
	Overweight	217(36.3)	69(31.8)	148(68.2)	
	Obese	37(6.2)	17(45.9)	20(54.1)	
Triglyceride	Yes	179(29.9)	73(40.8)	106(59.2)	0.005
creatinine	Yes	73(12.2)	28(38.4)	45(61.6)	0.058

Participants of the present study reported diabetes, among which notable findings emerged regarding health indicators. It was observed that the prevalence of high blood pressure (*P* = 0.012), obesity (*P* = 0.08), and high blood cholesterol (*P* = 0.02) exhibited a statistically significant increase among the female participants, underscoring the sex-based disparities in these health parameters. Male participants had higher creatinine levels based on sex (67.9%) and family history of DM (78.6%) than females in both cases. Similarly, significantly higher blood pressure (*p* = 0.0003), cholesterol (*p* = 0.01), and triglycerides (*p* = 0.02) were reported in females with a family history of DM compared with male participants. (Table 2).

**Table 2:** The prevalence of risk factors of the type-2 diabetes mellitus by gender and Family history of DM

Item	Gender			Family history of DM		
	Male	Female	*p*-value	Male	Female	*p*-value
High blood Pressure	39(44.8)	48(55.2)	0.012	41(38.3)	66(61.7)	0.0003
smoking	37(97.4)	1(2.6)	0.41	34(89.5)	1(2.6)	0.38
Underweight	8(72.7)	3(27.3)	0.0062	7(63.6)	4(36.4)	0.0001
Normal	42(65.6)	22(34.4)	0.0001	37(57.8)	27(42.2)	0.007
Overweight	33(47.8)	36(52.2)	0.18	40(58.0)	29(42.0)	0.05
Obese	7(41.2)	10(58.8)	0.08	9(52.9)	8(47.1)	0.09
cholesterol	29(39.7)	44(60.3)	0.02	32(43.8)	41(56.2)	0.01
physically active	25(65.8)	13(34.2)	0.008	16(42.1)	22(57.9)	0.012
Triglyceride	43(58.9)	30(41.1)	0.09	30(41.1)	43(58.9)	0.02
Creatinine	19(67.9)	9(32.1)	0.003	22(78.6)	6(21.4)	0.003

The logistic regression analysis revealed compelling associations between various factors and the development of Type 2 Diabetes Mellitus (T2DM), as detailed in Table 3. These significant predictors included age, high blood pressure, obesity, abdominal obesity, high blood cholesterol, triglyceride levels, and family history, affecting both sexes. Delving deeper into the findings through multivariate logistic regression, we uncovered intriguing patterns. Notably, elderly male patients displayed a greater predisposition to T2DM, with an odds ratio (OR) of 2.1 and a *p*-value of 0.003, compared to their female counterparts in the same age bracket. Furthermore, within the male population aged 60 or older, the presence of a family history of DM accentuated this risk, resulting in a striking 3.1 times higher prevalence compared to females, who exhibited a 2.4 times higher risk (OR = 2.4, *p* = 0.0008). In rural areas, female patients exhibited a higher prevalence of DM (OR = 1.7, *p* = 0.016) in comparison to their male counterparts (OR = 1.42, *p* = 0.15). Moreover, among female patients residing in rural areas who also had a family history of DM, a notably higher prevalence was observed (OR = 2.2, *p* = 0.007) when compared to males within the same cohort. Females were 1.67 times more likely to develop diabetes, who did not participate in physical activity (OR = 1.67, *p* = 0.003) compared with male (OR = 1.4, *p* = 0.09). Men with high blood pressure showed a strong association with T2DM (OR = 2.4, *P* = 0.0002) and women with high blood pressure (OR = 1.8, *P* = 0.008). In our study, we observed a notably elevated prevalence of comorbid high blood pressure among male patients with a family history of diabetes. This was associated with an odds ratio (OR) of 2.3 and a *p*-value of 0.0001. Moreover, our investigation revealed another intriguing finding: male patients with diabetes exhibited markedly elevated cholesterol levels, with an OR of 1.9 (*p* = 0.001). This intriguing relationship between diabetes and cholesterol levels was not limited to sex. Both male (OR = 2.47, *p* = 0.0001) and female (OR = 2.1, *p* = 0.0004) diabetes patients displayed highly significant associations with cholesterol levels who had a family history of DM. The risk of T2DM was significantly associated with triglycerides in both sexes (1.58 times higher in males, and 1.71 times higher in females). In more men, obesity was associated with a higher chance of type 2 diabetes (OR 1.8, *p* = 0.0052).

**Table 3:** The odds ratio of risk factors for developing T2DM by gender and Family History

		Gender				Family history			
		Male		Female		Male		Female	
Factor	categories	OR (95% CI)	*p*-value*		*p*-value*	OR (95% CI)	*p*-value*	OR (95% CI)	*p*-value*
Age	18–39	Ref. 1		Ref. 1		Ref. 1		Ref.1	
	40–49	1.3(0.81–1.9)	0.28	1.5(0.99–2.3)	0.27	1.13(0.91–1.4)	0.39	1.6(1.1–2.1)	0.01
	50–59	1.7(1.02–2.6)	0.06	1.63(1.1–2.1)	0.05	1.9(1.5–2.3)	0.004	2.5(1.8–3.2)	< 0.0001
	60 or older	2.1(1.3–2.8)	0.003	1.8(1.3–2.6)	0.04	3.1(2.4–3.6)	< 0.0001	2.4(1.7–3.2)	0.0008
Residency	Urban	Ref. 1		Ref. 1		Ref. 1		Ref.1	
	Rural	1.42(0.8–2.32)	0.15	1.7(1.1–2.6)	0.016	1.2(0.9–1.6)	0.27	2.2(1.7–2.7)	0.007
Education level	Illiterate	1.3(0.8–1.9)	0.18	2.3(1.5–2.9)	0.0001	2.0(1.3–2.8)	0.009	3.6(2.6–4.2)	< 0.0001
	Primary school	1.5(1.0- 2.3)	0.05	2.0(1.2–2.7)	0.007	1.8(1.2–2.6)	0.003	3.0(2.2–4.01)	< 0.0001
	Secondary	1.2(0.7–1.7)	0.26	1.3(0.82–1.7)	0.06	1.9(1.4–2.88)	0.0005	2.1(1.67–2.7)	0.0001
	Graduate	1.3(0.75–1.9)	0.31	1.42(0.93–2.1)	0.17	1.8(1.36–2.41)	0.002	1.3(0.97–1.6)	0.19
	Post-graduate	Ref. 1		Ref. 1		Ref. 1		Ref.1	
Physically active	Yes	Ref. 1		Ref. 1		Ref. 1		Ref.1	
	No	1.4(0.8–1.93)	0.09	1.67(1.1–2.4)	0.003	1.7(1.1–2.7)	0.02	2.86(2.13–3.8)	< 0.0001
High blood pressure	Yes	2.4(1.56–3.1)	0.0002	1.8(1.3–2.7)	0.008	2.3(1.7–2.9)	0.0001	1.9(1.36–2.7)	0.002
	No	Ref. 1		Ref. 1		Ref. 1		Ref.1	
Cholesterol	Yes	1.9(1.4–2.6)	0.001	1.52(1.2–2.1)	0.03	2.47(1.8–3.2)	< 0.0001	2.1(1.38–2.9)	0.0004
	No	Ref. 1		Ref. 1		Ref. 1		Ref.1	
Smoking	Yes	0.94(0.4–1.2)	0.36	0.72(0.3–0.94)	0.65	1.4(1.1–2.3)	0.09	1.1(0.8–1.5)	0.31
	No	Ref. 1		Ref. 1		Ref. 1		Ref.1	
Triglycerides	Yes	1.58(1.2–2.09)	0.04	1.71(1.36–2.21)	0.01	1.6(1.3–2.2)	0.04	1.92(1.5–2.6)	0.007
	No	Ref. 1		Ref. 1		Ref. 1		Ref.1	
BMI	Underweight	0.93(0.62–1.2)	0.17	0.88(0.59–1.1)	0.31	1.2(0.83–1.6)	0.19	1.0(0.62–1.3)	0.31
	Normal	Ref. 1		Ref. 1		Ref. 1		Ref.1	
	Overweight	1.3(0.9–1.7)	0.18	1.1(0.84–1.4)	0.21	2.2(1.7–2.9)	0.001	1.4(1.1–2.03)	0.05
	Obese	1.8(1.3–2.3)	0.0052	1.52(1.2–1.9)	0.008	3.6(2.3–4.8)	< 0.0001	1.8(1.3–2.6)	0.0015
Creatinine	Yes	2.1(1.6–2.8)	0.0001	1.4(1.09–2.2)	0.07	4.1(2.8–5.8)	< 0.0001	2.6(1.7–3.9)	< 0.0001
	No	Ref. 1		Ref. 1		Ref. 1		Ref.1	

*Multiple logistic regression models. BMI: Body mass index. OR = Odds Ratio, CI = confidence interval. Significant level - *p* < 0.05

## Discussion

The findings of this study shed light on several crucial aspects of T2DM prevalence within the surveyed population. Notably, a higher prevalence of T2DM was observed among female participants (31.3%) compared to their male counterparts (23%). This sex disparity in T2DM prevalence is consistent with previous studies highlighting potential sex-specific variations in diabetes susceptibility and risk factors [24–26]. Furthermore, our investigation revealed a noteworthy age-related trend in T2DM prevalence, with the highest rates observed in individuals aged 60 years and above, reaching a substantial 44.3%. This finding aligns with the well-established understanding that T2DM risk increases with age, underscoring the importance of age-specific diabetes prevention and management strategies [27, 28]. Intriguingly, our study uncovered a statistically significant association (*p* = 0.05) between education levels and T2DM prevalence, indicating that a higher prevalence of T2DM was evident among individuals with lower educational attainment, such as those who were illiterate or had only completed primary education. This correlation between educational attainment and T2DM risk underscores the potential influence of socio economic factors on diabetes prevalence and emphasizes the need for targeted health literacy initiatives [18, 27, 29].

We identified several lifestyle and health-related factors as significant contributors to the prevalence of T2DM in our comprehensive analysis of the study cohort. A complex interplay between various risk factors and diabetes occurrence is revealed by these findings. First of all, 15.2% of the participants in our study were smokers. Smoking has long been recognized as a harmful lifestyle choice associated with a higher risk of numerous health conditions, including type 2 diabetes [30, 31]. Smoking is not directly associated with diabetes prevalence in our study, but smoking may be a risk factor for T2DM due to its adverse health effects. In our study, 62.2% of participants reported a family history of diabetes. There is established literature suggesting a genetic predisposition to T2DM [21, 32, 33]. There was a statistically significant correlation (*p* = 0.004) between individuals with a family history of diabetes and the prevalence of diabetes within this group, with 28.8% affected. Individuals with a familial predisposition to T2DM should be targeted for prevention strategies because genetic factors play a significant role in risk. Regular physical activity is a well-known protective factor against T2DM [33, 34]. Our analysis revealed that 18.9% of the participants engaged in regular physical activity. Intriguingly, while a majority of these physically active individuals did not have diabetes, 33.6% of those who maintained an active lifestyle also exhibited signs of diabetes. This finding emphasizes the multifaceted nature of diabetes risk and suggests that physical activity alone may not mitigate risk in some individuals. Other factors, such as genetics and dietary choices, likely contribute to this complex relationship. We meticulously monitored the blood pressure of 32.8% of the study participants and assessed cholesterol levels in 35.3% of the cohort. Hypertension and dyslipidemia have been associated with T2DM [35, 36]. Our data provide valuable insights into the prevalence of these risk factors within the study population and their potential role in T2DM risk.

Lastly, within our study cohort, a relatively small subset comprising only 6.2% of participants met obesity criteria. Remarkably, 17% were diagnosed with T2DM. This finding underscores the strong association between obesity and T2DM, as excess body weight is a well-established risk factor for diabetes [37–40]. It reinforces the importance of obesity prevention and management as a critical component of diabetes prevention efforts.

Female participants were statistically significantly more likely to have high blood pressure (*P* = 0.012), obesity (*P* = 0.04), and high blood cholesterol (*P* = 0.02) than their male counterparts. According to previous research [28, 41], these health parameters affect diabetes outcomes differently according to sex [18, 41]. In light of these findings, it is important for interventions to take sex-specific risk factors into consideration. Additionally, we found that females with a family history of diabetes exhibited significantly higher levels of blood pressure (*p* = 0.0003), cholesterol (*p* = 0.01), and triglycerides (*p* = 0.02) than males with similar family histories. Accordingly, genetic predisposition may impact metabolic parameters differently in women, making further study of the mechanism necessary. Interestingly, male participants consistently exhibited higher creatinine levels both based on sex (67.9%) and family history of diabetes (78.6%) than their female counterparts. Elevated creatinine levels may indicate impaired kidney function, a common complication of diabetes [42]. Sex-related creatinine levels emphasize the importance of kidney health assessment in diabetes management, especially among male individuals.

The observation of a higher predisposition to T2DM among elderly male patients, as evidenced by an odds ratio (OR) of 2.1 and a *p*-value of 0.003 when compared to their female counterparts in the same age group, sheds light on an important aspect of T2DM epidemiology. This finding underscores the complex interplay of age and sex in diabetes risk and has significant clinical implications. Age is a well-established risk factor for T2DM, with advancing years often associated with an increased likelihood of developing the condition [43, 44]. Our study reaffirms this relationship, highlighting the vulnerability of elderly individuals to T2DM. However, the sex-specific disparity within this age group is a novel and crucial finding. Several factors may contribute to the increased T2DM risk in elderly males compared to their female counterparts. Hormonal differences between sexes, particularly in the postmenopausal period for women, could influence metabolic parameters and insulin sensitivity [27, 28, 45]. Additionally, lifestyle and behavioral factors, such as dietary choices and physical activity patterns, may differ between elderly men and women, impacting diabetes risk [46–48]. We observed sex-specific differences in this association, shedding light on the complex interplay between hypertension and diabetes. Males with high blood pressure exhibited a particularly strong association with T2DM, with an OR of 2.4 and a highly significant *p*-value of 0.0002. This finding aligns with extensive literature highlighting the bidirectional relationship between hypertension and T2DM, where one condition can contribute to the development and exacerbation of the other [46, 49]. It emphasizes the importance of comprehensive cardiovascular risk assessment in individuals with T2DM, particularly in males. This finding suggests that a family history of diabetes may compound the risk of hypertension in males, emphasizing the importance of considering genetic and familial factors in cardiovascular risk assessment and management strategies. The findings regarding the associations between diabetes and lipid profiles, specifically cholesterol and triglyceride levels, as well as the relationship between obesity and T2DM, offer valuable insights into the multifaceted nature of diabetes risk factors and have important clinical implications. Firstly, our study observed markedly elevated cholesterol levels among male patients with diabetes, with an odds ratio (OR) of 1.9 and a significant *p*-value of 0.001. This underscores the well-established link between diabetes and dyslipidemia, particularly elevated levels of low-density lipoprotein cholesterol (LDL) [50, 51]. Dyslipidemia is a known risk factor for cardiovascular disease in individuals with diabetes and is an essential component to consider in diabetes management. Importantly, this relationship between diabetes and cholesterol levels was not limited to sex. Both male (OR = 2.47, *p* = 0.0001) and female (OR = 2.1, *p* = 0.0004) diabetes patients displayed highly significant associations with cholesterol levels. This suggests that addressing dyslipidemia should be an integral part of diabetes care for both men and women. Additionally, our study revealed that the risk of T2DM was significantly associated with elevated triglyceride levels in both sexes, with a 1.58 times higher risk in males and a 1.71 times higher risk in females. This reinforces the importance of monitoring triglyceride levels in individuals at risk for or diagnosed with diabetes, as high triglycerides were associated with insulin resistance and an increased risk of cardiovascular complications [52]. Furthermore, the association between obesity and an increased risk of T2DM, particularly in men (OR 1.8, *p* = 0.0052), highlights the critical role of weight management and lifestyle modifications in diabetes prevention. Obesity is a well-established risk factor for T2DM, as excess adiposity contributes to insulin resistance and systemic inflammation [53].

## Conclusions

In the current study, findings underscore the critical necessity for tailored diabetes prevention and management approaches that account for these varied factors to effectively combat the burden of T2DM within our population. Additionally, our research highlights the existence of sex-based disparities in health indicators among individuals with reported diabetes, emphasizing the need for targeted interventions. Notably, we observed elevated rates of comorbid high blood pressure among male patients with a family history of diabetes and a higher prevalence of certain conditions among females in rural areas. Furthermore, our findings stress the importance of considering lipid profiles, obesity, and their sex-specific associations in assessing and managing diabetes risk. Overall, our study contributes valuable insights to the field, providing a foundation for more nuanced and effective strategies in tackling the challenges posed by T2DM.

## Limitations

The study conducted by the authors in the Saudi Arabia has certain limitations that need to be considered when interpreting the research findings. One key limitation is the specific demographic variables that were considered in this study. The research focused only on participants who had specific characteristics, such family history of diabetes. We have a limited number of participants (sample size), so it is difficult to generalize the findings to all diabetic patients. The participants were selected from a specific geographical location only. The reliance on self-reporting may be prone to recall bias. Researchers should acknowledge these limitations when interpreting findings and drawing conclusions.

### Electronic supplementary material

Below is the link to the electronic supplementary material.

Supplementary Material 1

## Data Availability

Data is provided within the manuscript or supplementary information files. All data and materials utilized by our research team are exclusively our own. We do not utilize any third-party data.

## Abbreviations

USA: United States of America
PC: Primary care
OR: odds ratio
T2DM: Type 2 Diabetes Mellitus
WHO: World Health Organization
KSU: King Saud University
IRB: Institutional review board
SPSS: Statistical package for the social sciences
ESRD: End-stage renal disease
IDF: International Diabetes Federation
